# Eugenol: An Insight Into the Anticancer Perspective and Pharmacological Aspects

**DOI:** 10.1002/fsn3.70727

**Published:** 2025-08-03

**Authors:** Ahmad Mujtaba Noman, Muhammad Tauseef Sultan, Aimen Mazhar, Waqas Ahmad Khan, Muhammad Imran, Muzzamal Hussain, Ehab M. Mostafa, Ahmed H. El‐Ghorab, Mohammed M. Ghoneim, Samy Selim, Mohamed A. Abdelgawad, Entessar Al Jbawi, Suliman A. Alsagaby, Waleed Al Abdulmonem

**Affiliations:** ^1^ Faculty of Food Science and Nutrition, Department of Human Nutrition Bahauddin Zakariya University Multan Pakistan; ^2^ TIMES Institute Multan Pakistan; ^3^ Department of Food Science and Technology University of Narowal Narowal Pakistan; ^4^ Department of Food Science Government College University Faisalabad Faisalabad Pakistan; ^5^ Department of Pharmacognosy College of Pharmacy, Jouf University Sakaka Saudi Arabia; ^6^ Department of Chemistry College of Science, Jouf University Sakaka Saudi Arabia; ^7^ Department of Pharmacy Practice College of Pharmacy, AlMaarefa University Ad Diriyah Riyadh Saudi Arabia; ^8^ Department of Clinical Laboratory Sciences College of Applied Medical Sciences, Jouf University Sakaka Saudi Arabia; ^9^ Department of Pharmaceutical Chemistry College of Pharmacy, Jouf University Sakaka Saudi Arabia; ^10^ Sugar Beet Research Department Crops Research Administration, General Commission for Scientific Agriculture Research (GCSAR) Damascus Syria; ^11^ Department of Medical Laboratory Sciences College of Applied Medical Sciences, Majmaah University AL‐Majmaah Saudi Arabia; ^12^ Department of Pathology College of Medicine, Qassim University Buraidah Kingdom of Saudi Arabia

**Keywords:** breast cancer, eugenol, hypoglycemia, IL‐6, lung cancer, TNF‐α, toxicity

## Abstract

The aura of the 21st century has been transformed due to a revolutionized health care system and modern industrial applications. However, human health and well‐being are still a global challenge, affected by various metabolic and pathological morbidities, causing millions of mortalities. Cancer is one of the leading causes of fatalities globally, occurring due to pathogens, dietary factors, environmental toxins, occupational chemical exposure, and radiation. These factors lead to oxidative stress (OS), inflammation, DNA damage, epigenetic and genetic mutations, cell proliferation, tumorigenesis, and other chronic metabolic disorders such as diabetes mellitus, cardiovascular disorders, gastrointestinal problems, and hepato‐renal syndrome. Among various types of cancers, lung cancer is leading them all, followed by breast and colorectal cancer (CRC). The oncogenesis includes activation of pro‐inflammatory markers (IL‐6, IL‐1β, TNF‐α), resulting in overexpression of oncogenes, downregulation of tumor suppressor genes (TSGs) like p53, pRb, APC, PTEN, suppression of antioxidant enzymes (GSH, CAT, SOD), and activation of signaling pathways (PI3K/Akt/mTOR/NF‐κB, Ras/MAPK). However, plant‐based herbal medicine is a valuable, affordable, and available management approach to reduce the risks of oncogenesis and other ailments. The bioactive compounds of these herbs make them an effective way to reduce inflammation, cancer, and the risk of other health problems. Eugenol (Eug), a volatile phenolic bioactive compound with a formula of C10H12O2, has been reported to have anticancer, antidiabetic, cardio‐ and pulmonary protective roles. Moreover, it can improve gut health and prevent neurodegenerative disorders. According to WHO, the safe dose of eugenol is 2.5 mg/kg for consumption. The current review focuses on the anticancer potential and other pharmacological aspects of eugenol through possible mechanisms.

## Graphical abstract

Eugenol (Eug), a volatile phenolic bioactive compound with formula of C10H12O2 have been reported with anticancer, antidiabetic, cardio‐ and pulmonary protective role. Moreover, it has the ability to improve gut health and prevent neurodegenerative disorders. According to WHO, the safe dose of Eug is 2.5 mg/kg for consumption. The current review focuses on anticancer potential and other pharmacological aspects of eugenol through possible mechanisms.

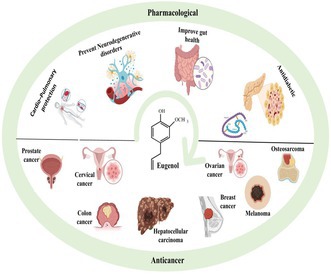

## 1 Introduction

The prevailing chronic ailments have seriously intimidated public health, demanding a multisectoral and interdisciplinary approach. The manifestation of the global disease burden alarming countries worldwide includes various root causes and risk factors such as irregular eating patterns, lifestyle alterations, over‐the‐counter (OTC) drugs, toxins, chemicals, and pathogenic disorders. Despite advanced technology and a revolutionized healthcare system, preventing and managing these chronic disorders remain a problem (Xavier et al. 2024). However, to manage these health issues, dietary management is a suitable and appropriate strategy due to availability and affordability. The plant‐based herbal medicine application is a centuries‐old cultural practice, and in recent decades, their demand has increased due to valuable bioactivities and minimal adverse effects. The herbal bioactive compounds have been acknowledged for their therapeutic potential and medicinal properties (Yang and Yang 2021). Eugenol, a phenolic bioactive compound with a formula of C_10_H_12_O_2_, also referred to as 4‐allyl‐2‐methoxyphenol, is a key phenylpropene, belongs to class phenylpropanoids, which are major constituents of essential oil. Eugenol is mainly found in several herbs, including *Myristica fragrans* , *Cinnamomum verum* , *C. loureirii*, *Ocimum gratissimum* , and *Ocimum basilicum* . It was isolated in 1929 for the first time, and its commercial production began during the early 1940s' in the United States. The maximum pure eugenol can be isolated through acids; however, alkalis are also used to obtain eugenolate compounds. It is a pale‐yellow substance with a lipophilic nature and is slightly soluble in water (Abdou et al. 2021).

Previous studies have reported anti‐inflammatory (Damasceno et al. 2024), hypotensive (Jandhyam et al. 2024), anticancer (Padhy et al. 2022), antioxidant (Candido Júnior et al. 2022), antiparasitic (Cheraghipour et al. 2023), antimicrobial (Di Consiglio et al. 2023), antiseptic (Marchese et al. 2017), dental analgesic (Shen and Yan 2024) activities of eugenol. Cancer is the leading cause of morbidities and fatalities globally, occurring due to overproduction or imbalance of reactive oxygen species (ROS) and reactive nitrogen species (RNS), which are highly unstable and lead to oxidative stress (OS) and inflammation. Numerous risk factors are inducing OS, thus triggering pro‐inflammatory markers (IL‐6, IL‐1β, TNF‐α, MDA), suppression of tumor suppressor genes (TSGs), activation of oncogenes (ERBB2, BCR/ABL1, K‐Ras, PIK3CA, NMYC), and epigenetic aberrations. Collectively, all these events lead to DNA damage and, ultimately, oncogenesis (Greten and Grivennikov 2019).

The current review focuses on the anticancer potential and other pharmacological aspects of eugenol, illuminating possible mechanisms. The current review is unique due to its clarity, specificity, and interdisciplinary appeal. By combining “Anticancer Perspective” with “Pharmacological Aspects,” the review highlights a dual focus on therapeutic potential and underlying biological mechanisms. This balanced scope enhances its relevance for cancer researchers and pharmacologists, making it both informative and compelling for a diverse scientific audience.

## 2 Methodology

The methodology section was designed to review the updated anticancer properties and other health benefits of eugenol, and relevant peer‐reviewed articles were retrieved from databases and search engines such as Google Scholar, PubMed, Science Direct, Scopus, and Web of Science, along with various keywords like eugenol bioavailability, eugenol anticancer, eugenol antidiabetes, eugenol cardioprotection, eugenol and gut health, eugenol toxicity, and eugenol food applications. Boolean operators such as (AND, OR) were used to increase the search. Inclusion criteria: original research articles with high citations, covering eugenol's anticancer role, other pharmacological aspects, and applications. Exclusion criteria: non‐English articles and duplicate articles. The preclinical findings were systematically analyzed to evaluate the therapeutic potential of eugenol.

## 3 Bioavailability of Eugenol

Digestibility and bioavailability are two major factors affecting the health‐promoting benefits of bioactive compounds and other ingredients. The bioavailability of compounds mainly depends on pharmacodynamics and pharmacokinetics, and knowledge of these mechanisms develops a better understanding of the proper pharmacological aspects of that specific compound. Studies on the pharmacodynamics and pharmacokinetics of eugenol demonstrated that its bioavailability is poor; however, chemical configuration modification and application of other methods can improve its bioavailability. The hydrophobic nature of eugenol makes it clear that it has better absorbance in other solvents than aqueous media (Joardar et al. 2020). Furthermore, multiple carriers like liposomes, glycodendritic polyamine dextran, solid lipid nanoparticles, and corn protein nanoparticles have been reported to deliver eugenol. Cyclodextrins (CDs) are more suitable for eugenol delivery due to their unique structure, as CDs are oligosaccharides prepared by specific enzyme treatments such as *Bacillus macerans* on starch. Structurally, CDs are doughnut‐shaped constituents with two faces and exist in three types with 6–8 glucose molecules joined via 1,4‐α linkage. The basic face is narrow and contains primary hydroxyl groups, while the other consists of a hydrophobic inner part and secondary hydroxyl groups (Jansook et al. 2018).

## 4 Biodegradation and Elimination

Eugenol is volatile in nature; therefore, to attain its maximum advantages, it should be obtained in powder or solidified form. Porous silica is another suitable candidate for solidification and better eugenol delivery. Yao et al. (2024) prepared eugenol‐porous silica powder and determined its bioavailability. The results showed that porous silica effectively solidifies eugenol at lower dosages and improves eugenol release in vitro and in vivo. The half‐life of eugenol was also extended, which may be associated with its adsorption on porous silica. Xu et al. (2023) studied the pharmacokinetics and bioavailability of eugenol in carp fish and detected 0.01 μg/g eugenol in tissue and 0.008 μg/mL eugenol in plasma. The highest concentration was found in the liver, while the lowest was quantified in the muscle. They concluded that the half‐life of eugenol was reduced with an increase in concentration, and eugenol was eradicated rapidly in carp tissues. Previously, Wang et al. (2019) checked the bioavailability of microencapsulated eugenol in Beagle dogs' pallets and reported that eugenol improved 23.6 times compared with free eugenol. Meinertz et al. (2014) detected eugenol in rainbow trout, as the fish was exposed to 100 mg l^−1^ AQUI‐S 20E for 30‐, 60‐, 120‐, and 240‐min duration and found 50, 58, 54, and 62 μg g^−1^ concentrations in fillet tissues.

Eugenol can be manufactured synthetically and biosynthetically by guaiacol allylation with allyl chloride and microbes, that is, *E. coli* , *B. cereus, and Corynebacterium* spp., respectively (Abrahão et al. 2013). Several organs quickly absorb it and then metabolize it in the liver, with 95% of the amount being excreted within 24 h. Due to its vulnerability and volatility, the encapsulation of eugenol is a more appropriate method to prevent its early absorption and enhance its stability, solubility, and activity (Mak et al. 2019). Guenette et al. (2006) investigated eugenol pharmacokinetics in male Sprague Dawley rats and found eugenol glucuronide and sulfate conjugates in the urine of rats. Eugenol (150 mg) in gelatin capsules was orally administered in healthy adults and absorbed very quickly, and ~55% is eliminated in urine after being transformed to glucuronic acid or eugenol sulfate conjugate in the liver (Fischer et al. 1990). Hou et al. (2021) investigated the elimination time of eight volatile constituents in rats and reported that eugenol was the first among the rapidly eliminated. However, elimination time can be enhanced by oxidation.

## 5 Antioxidant Potential

The antioxidant potential of the compound is the ability to scavenge ROS and RNS, thus preventing OS, inflammation, and cancer. ROS, RNS, OS, and inflammation alter molecular mechanisms, thus damaging vital organs. The studies on eugenol have proved its antioxidant and anti‐inflammatory properties. Mateen et al. (2019) reported that eugenol alleviated arthritis via attenuating pro‐inflammatory cytokines (TNF‐α, IL‐6, IL‐10). Jabbari et al. (2020) stated the anti‐arthritic activity of eugenol‐chitosan nanoparticles (Eug‐ch NPs) via reducing TGF‐β, CCL2/MCP‐1 gene expression, and MDA and FOXO3 levels. Eugenol (2.5, 5, 10 mg/kg) improved GSH, GPx, and CAT levels while reducing carrageenan‐induced OS in arthritic rats (Adefegha et al. 2019). Bittencourt‐Mernak et al. (2021) reported the anti‐inflammatory potential of eugenol against LPS‐induced acute lung injury via modulating IL‐6, IL‐1β, iNOS, MMP‐9, TIMP‐1, Jc‐Jun‐NH2, and MAPKs. Oroojan et al. (2020) reported the antioxidant activity of eugenol (50, 100, 200 μM) against H_2_O_2_‐induced OS in islets of Langerhans of rodents. They reported reduced MDA and improved SOD, CAT, and TAC levels. Di Consiglio et al. (2023) studied the antioxidant potential of eugenol‐hydroxyethyl methacrylate polymers. They reported that the presence of a phenol group in the side chain of polymers provides antioxidant and antimicrobial activity to this compound.

The gels and creams are effective methods to treat skin disorders, and the bioactive compounds in these products enable them to prove efficacious against a broad range of fungal infections and other skin diseases. Makuch et al. (2021) investigated the percutaneous absorption, retention, and antioxidant capacity of eugenol and eugenol derivatives based on gel and cream. They concluded that gel is a more appropriate vehicle for antioxidant activity and skin absorption. Aara et al. (2020) determined the antioxidant potential of eugenol and *piper betel* leaf extract, resulting in IC50 values of 114.34 ± 0.46 and 306.44 ± 5.28 for both specimens, respectively. Ekinci Akdemir et al. (2019) examined the antioxidant and antiapoptotic potential of eugenol against cisplatin‐induced testicular injury in a rat model. They found increased antioxidant enzymes, reduced lipid peroxidation, reverted histopathological changes, and overall reduced OS and apoptosis.

## 6 Anticancer Perspectives

**FIGURE 1:**
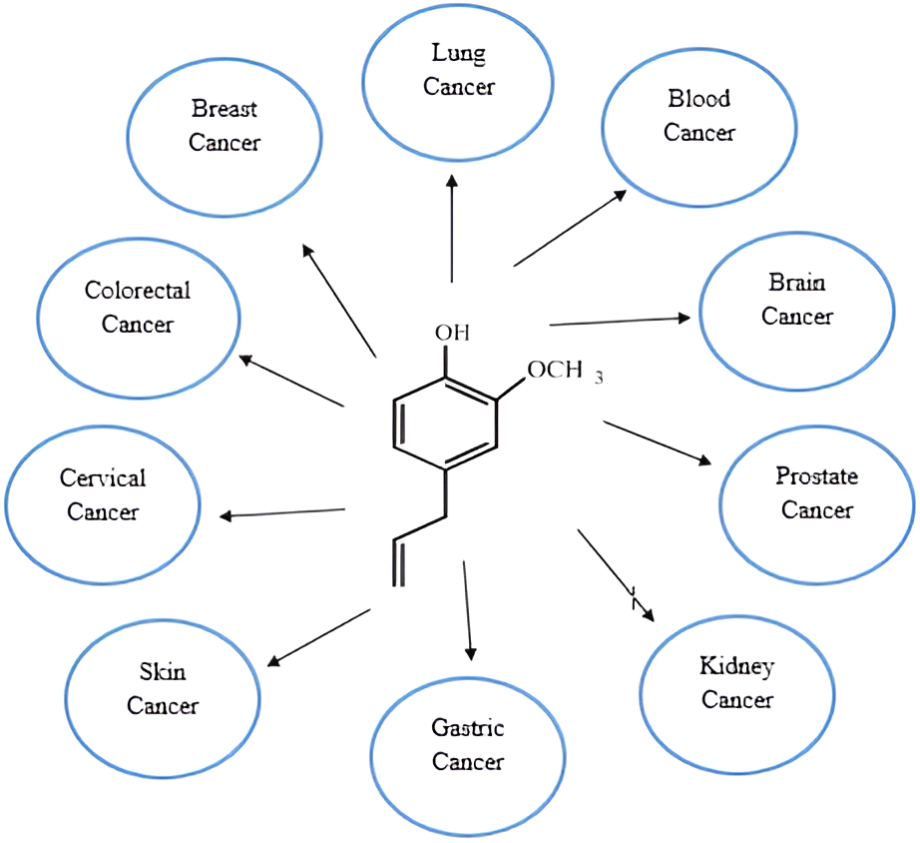
Anticancer potential of eugenol against various cancer types.

**FIGURE 2:**
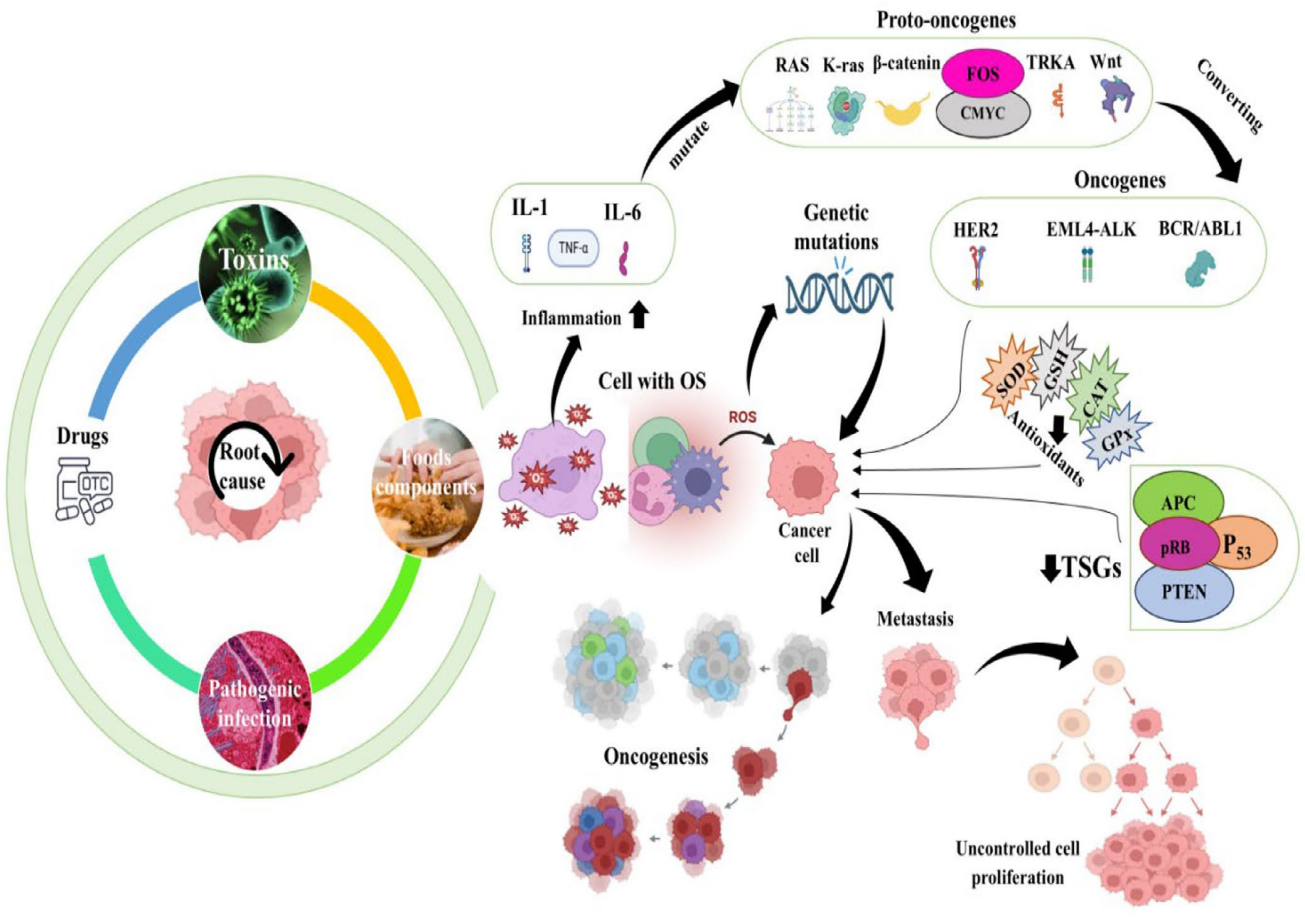
Inflammation and cancer mechanism through activation of pro‐inflammatory markers, genetic mutations conversion of proto‐oncogenes to oncogenes, reduction of the antioxidant enzyme system, and suppression of TSGs.

The rapid cancer progression and prevalence are increasing every day, chronically affecting individuals in both developed and developing nations. Numerous factors, such as unhygienic dietary intake, polluted environmental situations, and pathogenic diseases, contribute significantly to oncogenesis. Inflammation, OS, DNA damage, and genetic mutations are events that occur in cancer incidence. However, proper dietary strategies and an integrative approach can control and minimize the chronic condition. Plant‐based bioactive compounds are natural compounds that can potentially decrease the risk factors of cancer development (Maaz et al. 2025). The current part focuses on the anticancer potential of eugenol with possible biochemical mechanisms. The anticancer potential of eugenol against various cancer types is illustrated in Figure 1. In contrast, inflammation and cancer mechanisms via triggering of pro‐inflammatory markers, conversion of proto‐oncogenes to oncogenes, reduction of the antioxidant enzyme system, and suppression of TSGs are shown in Figure 2.

### 6.1 Lung Cancer

Lung cancer (LC) is a leading cancer that causes millions of morbidities and mortalities globally, including small cell lung cancer (SCLC) and non‐small cell lung cancer (NSCLC). According to the International Agency for Research on Cancer (IARC), the morbidities and causalities from LC in 2022 were 2,480,675 and 1,817,469, respectively. Smoking is a major risk factor responsible for ~85% of cases, while occupational chemical and heavy metal exposure, family history, and air pollution are also associated with LC (Thandra et al. 2021). Genetic alterations due to smoking can lead to dysplasia of the lung epithelium, and it may affect protein synthesis. Genetic changes in MYC, BCL2, and p53 genes are responsible for causing SCLC, and NSCLC involves p16, EGFR, and KRAS gene mutations. Furthermore, the dysfunction of p107, p130, PTEN, chromatin regulator CREBBP, and NOTCH receptors contributes to cancer progression (Lindeman et al. 2018).

Growth factors and angiogenesis significantly contribute to cancer development, as cancer cells release chemical signals that promote the development of new blood vessels to provide cancer cells with nutrients and oxygen. Su et al. (2024) studied the anticancer activity of eugenol against A549 cells and concluded that eugenol subdued cell migration and invasion by suppressing angiogenesis‐related protein expression and modulating JAK2/STAT3 pathways. Morsy et al. (2023) investigated eugenol's mitigated effect against diethyl nitrosamine‐induced LC in Wistar rats and concluded that eugenol (20 mg/kg bw) reduced LPO levels, inhibited TNF‐α, IL‐1β, improved GSH, GPx, SOD, Nrf2, downregulated NF‐κB p65, MCP‐1, Bcl‐2 expression, and elevated Ki‐67 levels. Choudhury et al. (2021) reported eugenol's anticancer potential against NDEA‐induced lung carcinogenesis in mice and found that eugenol significantly reduced Wnt/β‐catenin expression and reduced other markers like CD44, Notcht1, EpCAM, and Oct4. Cui et al. (2019) stated that eugenol inhibited tumor progression by suppressing p65 and TRIM59 expression and modulating the NF‐κB pathway in H1975 cells of the xenograft model. Eugenol significantly reduced cell proliferation and migration in A549 cell lines via modulating MMP‐2 and PI3K/Akt pathways (Fangjun and Zhijia 2018).

### 6.2 Breast Cancer

Breast cancer (BC) is the second most prevalent cancer among different cancers worldwide, and according to WHO, 2.3 million women were identified with BC, and 670,000 deaths were reported due to BC in 2022 (Nardin et al. 2020). Gender, hormonal changes, age, physiological conditions, and dietary and lifestyle behaviors are accounted for BC. However, the hormonal imbalance has a notable contribution to cancer development. Estrogen, an important hormone, is linked with an augmented risk of cancer progression. Moreover, hormonal replacement therapy (HRT) is also a key risk factor for BC (Dall and Britt 2017). Almost 5%–10% of cases of BC are due to genetic mutations, and BRCA1 and BRCA2 are important genes associated with cancer progression. BRCA1 and BRCA2 genes, positioned on chromosomes 17 and 13, respectively, are suppressor genes tangled in genomic stability, encrypt nuclear protein, and repair double DNA strand breaks. Some other genes like CHEK2, ATM, PALB2, and BRIP1 show less tendency to BC cases. However, patients with these gene mutations have 2–3 times a higher risk of developing malignant tumors (Chamseddine et al. 2022).

Eugenol has proved effective against breast cancer, as eugenol‐loaded nanoemulsions (150, 300, 600 μg/mL) proved efficacious against MCF‐7 breast cancer cells (Velho et al. 2024). Pangesti et al. (2024) reported the anticancer activity of eugenyl salicylate and isoeugenyl salicylate against breast cancer, and in silico screening demonstrated that both derivatives have the best binding affinity in MMP9. Sihombing and Arsianti (2024) investigated eugenol in estrogen receptor‐positive breast cancer via molecular docking and network pharmacology. The study concluded that eugenol acted on CASP3, EGFR, and PARP1 pathways, and docking showed that eugenol has the strongest binding with CASP3, followed by EGFR. The anticancer potential of eugenol against breast cancer via possible mechanisms is demonstrated in Table 1.

**TABLE 1:** Anticancer potential of eugenol against breast cancer via possible mechanisms.

	Invitro	Mechanism	References
Eugenol	MDA‐MB‐231, MCF‐7	cell cycle at G2 and S phase	(Alam 2023)
	Triple‐negative breast cancer	**↓**cell proliferation and metastasis, modulates NOD1‐NF‐κB	(Shi et al. 2023)
	Breast cancer receptors	**↓**Erα, mTOR, HSP90, ERBB2	(Rasul et al. 2022)
	MDA‐MB‐231, SK‐BR‐3	**↑**AKT, FOXO3a, Caspase‐3/9, p21	(Abdullah et al. 2021)
	MCF‐7	**↑**Caspase 3, **↓**TNF‐α, CK7 LC3BI/II ratio	(Fouad et al. 2021)
	CAFs	**↓**DNMT1 and DNMT3A	(Al‐Kharashi et al. 2021)
	MDA‐MB‐468	**↓**cell proliferation	(Valizadeh et al. 2021)

### 6.3 Colorectal Cancer

Colorectal cancer (CRC) has been increasing every day, with 19,26,425 new cases of colorectal cancer reported, thus making it the third leading cancer globally (IARC 2022). The formation of tiny cell groups (polyps) inside the colon transforms into tumors in the next 5–10 years, leading to CRC development. Congenital disorders, genetic alterations, inflammatory bowel illness, and other malignancies are risk factors for CRC (Al‐Muswie et al. 2023). CRC develops due to epigenetics and genetic alterations at microRNAs (miRNAs), which influence cancer‐associated pathways at the posttranscriptional level and thus contribute to CRC progression, metastasis, and amendments in oncogenes. The CRC pathways include CIN, MSI, and CIMP; CIC is responsible for ~80%–85% of CRC cases. CIC initiates growth‐promoting and diminished apoptotic pathways (Fischer et al. 2019). The APC, a TSG, is usually changed in colorectal cancers, and this mutation activates the Wingless/Wnt pathway. This Wnt signaling pathway further transforms KRAS and TP53, leading to the development of polyp cells to cancer, followed by TGF‐β1‐mediated cell signaling pathway and accelerated CRC development. The majority of CRC cases are due to altered KRAS and B‐Raf, which activate WNT‐APC‐CTNNB1, TGFB1‐SMAD, and RAS–RAF–MAPK pathways and promote proliferation with suppressed apoptosis (Ahronian et al. 2015).

CRC development involves the stimulation of molecules and proteins, which further leads to epigenetics and genetic changes; therefore, molecular‐level suppression is an effective approach to inhibit cell proliferation and invasion. Trivedi et al. (2023) investigated the anticancer effect of eugenol and β‐caryophyllene via network pharmacology against HCT116 cell lines. The C‐D‐T network revealed that eugenol significantly reduced the expression of CRC proteins, including HSP90AA1, CASP3, IGF‐1R, and ESR1. In addition, the molecular docking showed eugenol suppressed HSP90AA1 more efficiently than others. Ghodousi‐Dehnavi et al. (2021) studied the eugenol mitigation effect in HT‐29 CRC cell lines by modifying APC, p53, and KRAS. They found that eugenol (500 *μ*M) enhanced APC and p53 expression, while KRAS expression was substantially reduced. Apoptosis is a potent way to reduce tumorigenesis. However, cancer cells can replicate rapidly and can decrease apoptosis. Eugenol's ability to induce and enhance apoptosis in cancerous cells makes it a suitable strategy to alleviate CRC. Fadilah et al. (2017) investigated aryl eugenol, a derivative of eugenol, through interaction with Bcl‐2. They concluded that aryl eugenol enhances apoptosis and thus proved an effective candidate against oncogenesis. Nano‐delivery is another suitable strategy to prevent eugenol loss in the body, along with targeted delivery to the cancer site. Wijewantha et al. (2023) studied eugenol‐enzyme responsive nanoparticles efficiency against CRC, resulting in eugenol‐Nps reducing cell proliferation, migration, and invasion and inducing apoptosis.

### 6.4 Gastric Cancer

Gastric cancer (GC) prevalence is higher among males rather than females, affecting six of every 10 people above 65, and 9,68,784 new cases of stomach cancer and 6,60,175 deaths from GC have been reported in 2022 (IARC 2022). The main root cause of GC is dietary behavior along with lifestyle habits. The *H. pylori* infection, salty foods, obesity, smoking, alcohol, Epstein–Barr virus (EBV), nitroso compounds, low folate intake, and occupational exposures are the main risk factors of GC (Thrift and El‐Serag 2020). *H. pylori* is directly linked with gastric carcinoma, and a suitable environment with an impaired immune system of the host can manifold the risk of cancer rate. Mucosal damage through urease‐mediated myosin II stimulation due to urease, acetaldehyde protease, phospholipase, and ammonia secreted by this gram‐negative bacterium leads to cancer development. *H. pylori* is involved in the production of ROS and OS, leading to DNA damage via NF‐κB and Wnt/β‐catenin activation (Dincă et al. 2022). Overexpression of cell surface receptor c‐erbB2 of the tyrosine kinase family, altered K‐Ras oncogene, and irregularities in FGFR2/ErbB3/PI3 kinase pathway have been widely related to GC. Modifications in several growth factors involved in the GC progress further produce mediators that worsen the conditions. The augmented expression of TGFBR2, CDC25A, SMAD7, and RELA and downregulation of p27 are major events in cell proliferation and cancer invasion (Kumari et al. 2021). In addition, saturated NaCl (S‐NaCl) promotes the development of N‐methyl‐N′‐nitro‐N‐nitrosoguanidine, which induces gastric carcinomas (Balendra et al. 2023).

**FIGURE 3:**
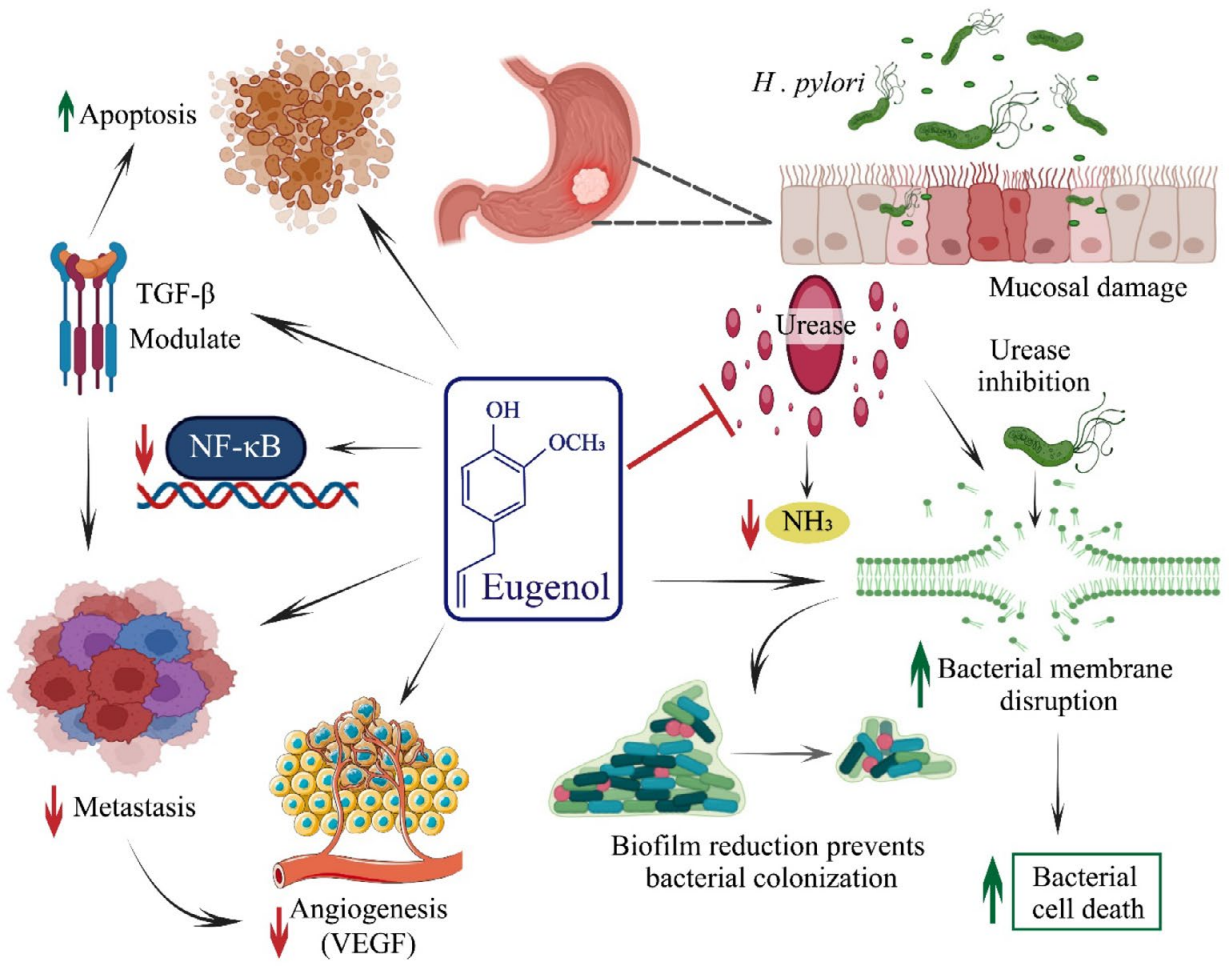
Anticancer potential of eugenol against gastric cancer through modulation of TGF‐β, downregulation of NF‐κB, induction of apoptosis, reduction of angiogenesis, inhabitation of metastatic cells, disruption of bacterial membrane, and bacterial cell death.

The TGF‐β signaling is a main player in GC metastasis, and the inhibition of the TGF‐β/SMAD4 pathway may prove a novel approach for therapeutic intervention in GC. Sarkar et al. (2020) investigated eugenol's anti‐metastatic potential through regulating TGF‐β signaling and reported suppressed cell proliferation and metastasis via modulating TGF‐β, independent of P21 and P53. Studies have proved that dysfunction of p53 results in cell proliferation because p53 is also responsible for cell division and apoptosis. Previously, Sarkar et al. (2015) studied the anticancer activity of eugenol via apoptosis induction in gastric cancer cells. They found that p53 is involved in apoptosis and reported enhanced apoptosis and improved caspase‐8/3 activity. Manikandan et al. (2011) reported that eugenol inhibited cell proliferation through NF‐κB suppression in a rat model GC induced by MNNG. The MNNG‐induced GC resulted from NF‐κB activation, IKKβ, cyclins, and PCNA upregulation, IκBα degradation, and p21, p53, and Gadd45 downregulation. However, eugenol administration reduced NF‐κB expression, inhibited cell proliferation, and modulated genes. Eugenol has been reported to reduce angiogenesis, inhibit invasion, and trigger apoptosis via modulating Bcl‐2, Apaf‐1, MMP 2/9, VEGF, TIMP‐2, VEGFR1, and RECK expression (Manikandan et al. 2010). The anticancer potential of eugenol against GC via apoptosis induction, metastasis inhibition, downregulation of NF‐κB, and angiogenesis reduction is shown in Figure 3.

### 6.5 Cervical Cancer

Cervical cancer (CC) is prevailing rapidly among women, with 660,000 new cases and about 350,000 expiries reported in 2022, thus making it the 4th most common cancer of females. Smoking, impaired immunity, multiple sex partners, and sexually transmitted pathogens are risk factors for CC. However, human papillomavirus (HPV) is known for more than 75% of CC cases, and the occurrence is high among women aged 35–44 and low in younger females (Zhang et al. 2020). Types 16 and 18 of HPV are most notorious for causing high‐grade CC. The E6 and E7 oncoproteins of HPV disrupt the host cell cycle, especially E6, which disturbs p53 and apoptosis signaling cascade proteins (Bak, FADD, procaspase 8), whereas E7 interacts with retinoblastoma protein (pRB). Alongside, the E5 protein may play a role in immune dysfunction and OS, and microRNAs also play a role in cervical carcinogenesis (Romero‐Masters et al. 2022).

Studies on eugenol have proved its effectiveness against CC through p53 regulation and apoptosis induction. Permatasari et al. (2021) investigated the antimetastatic effect of eugenol in HeLa cancer cells and reported the downregulation of Snail‐1 and vimentin expression, upregulation of E‐cadherin protein expression, and reduced cell migration. Fathy et al. (2019) reported eugenol's anticancer activity against HeLa cells through enhanced sensitivity towards cisplatin and radiation. They concluded improved caspase 3/9 activity, enhanced Bax and Cyt‐c expression, reduced expression of Bcl‐2, Cox‐2, and IL‐1β, and induced apoptosis. Das et al. (2018) studied the anticancer potential of eugenol in HeLa cell lines and reported that eugenol (200 mg/mL) inhibited cell multiplication and induced apoptosis. Hemaiswarya and Doble (2013) reported that eugenol (153 μM) combined with 5‐fluorouracil proved effective in inhibiting cell growth and division in HeLa cells. Similarly, eugenol (200–350 μM) with sulforaphane (6.5–8 μM) lowered the expressions of COX‐2, IL‐β, and Bcl‐2 and inhibited cell proliferation (Hussain et al. 2012).

### 6.6 Prostate Cancer

The prostate gland present below the bladder produces and forces semen through the urethra when on ejaculation and gets larger with age. The common prostate gland problems are prostate cancer, prostatitis, and benign prostatic hyperplasia (BPH). Prostate cancer (PC) is the 2nd most prevalent cancer among men, and according to IRAC, 2022, 14,67,854 new cases and 3,97,430 deaths from prostate cancer have been reported, which means 1 in 8 men can be identified with PC during their lifetime (Gandaglia et al. 2021). Prostatic intraepithelial neoplasia (PIN) and androgenic regulation of prostate cancer are vital processes responsible for PC progression. Androgenic regulators (ARs) are crucial transcription factors in cancer development through nuclear translocation of the receptors, proliferation, cell differentiation, and apoptosis (Dahiya and Bagchi 2022). Growth factors like TGF‐β, IGF, EGF, and FGF are ARs dependent, and EGF, with its membrane‐related tyrosine receptor kinase EGF‐1, is responsible for the progress of cancer cells by enhanced migration. Moreover, TSG, like PTEN, negatively modulates the PI3K/AKT/mTOR pathway and stumbles the cell cycle at the G1 stage, causing cell proliferation. Thus, the dysfunction of PTEN outcomes in an upsurged PI3K/AKT/mTOR pathway and blighting normal AR regulation, subsequent amplified proliferation, and declined apoptosis. Moreover, serine/threonine (PIM‐1), a proto‐oncogene, is vital in cell proliferation, and studies have proved that PIM‐1 kinase is involved in cellular development, immunoregulation, and oncogenesis; therefore, it is an appropriate therapeutic target for PC (Imada et al. 2021).

The deficiency of treatment strategies has limited cancer management. However, an integrative approach or combination therapy proved more effective than a single management strategy. Previously, Ghosh et al. (2009) investigated the combined effect of eugenol and 2‐methoxy estradiol (2‐ME2) against PC and reported cell inhibition, cell apoptosis, and cell cycle arrest. In addition, combined therapy enhanced Bax expression to induce apoptosis and suppressed Bcl‐2. Radiotherapy, along with bioactive compounds, could be a valuable cancer management approach. The radioiodinated eugenol has proved efficient in inhibiting cell proliferation and thus proving an effective candidate in PC3 adenocarcinoma (Dervis et al. 2017).

### 6.7 Skin Cancer

Skin is the largest organ in the body; protecting organs, protecting temperature regulation, water balance, sensation, and vitamin D synthesis are basic functions of skin. It consists of three layers: epidermis, dermis, and hypodermis, with different anatomical structures and functions. Skin cancer (SC) has several types, but melanoma is the most critical and originates from melanocytes, accounting for 75% of skin cancer fatalities. Approximately 3,31,722 new cases and 58,667 deaths were reported in 2022 in both genders (IRAC 2022). Sun and UV light exposure, age, skin type, genetics, and immunodeficiency are major risk factors involved in melanoma (Dzwierzynski 2021). A series of pathways with genetic mutations is involved in the pathophysiology of melanoma. The mutation of BRAF, a proto‐oncogene, particularly the V600E mutation, is responsible for 40%–60% of melanoma cases and is associated with sun exposure and genomic instability. BRAF encodes a serine/threonine protein kinase as part of the RAS–RAF/MEK–ERK kinase pathway, which promotes cell proliferation (Wen et al. 2024). The NRAS‐mutation pathway is another pathway of melanoma, leading to MAPK pathway activation, and mutations are generally found in Q60/61 and G12/13 codons. Like the RAS–RAF/MEK–ERK pathway, the PI3K‐AKT/PTEN pathway is also involved in cell proliferation and plays a crucial role in melanoma. Moreover, oncogene Cyclin‐dependent kinase 4 (CDK4) and TSG CDKN2A, which encodes p16INK4a, have been linked with familial melanoma progress (DeLeon et al. 2020).

**FIGURE 4:**
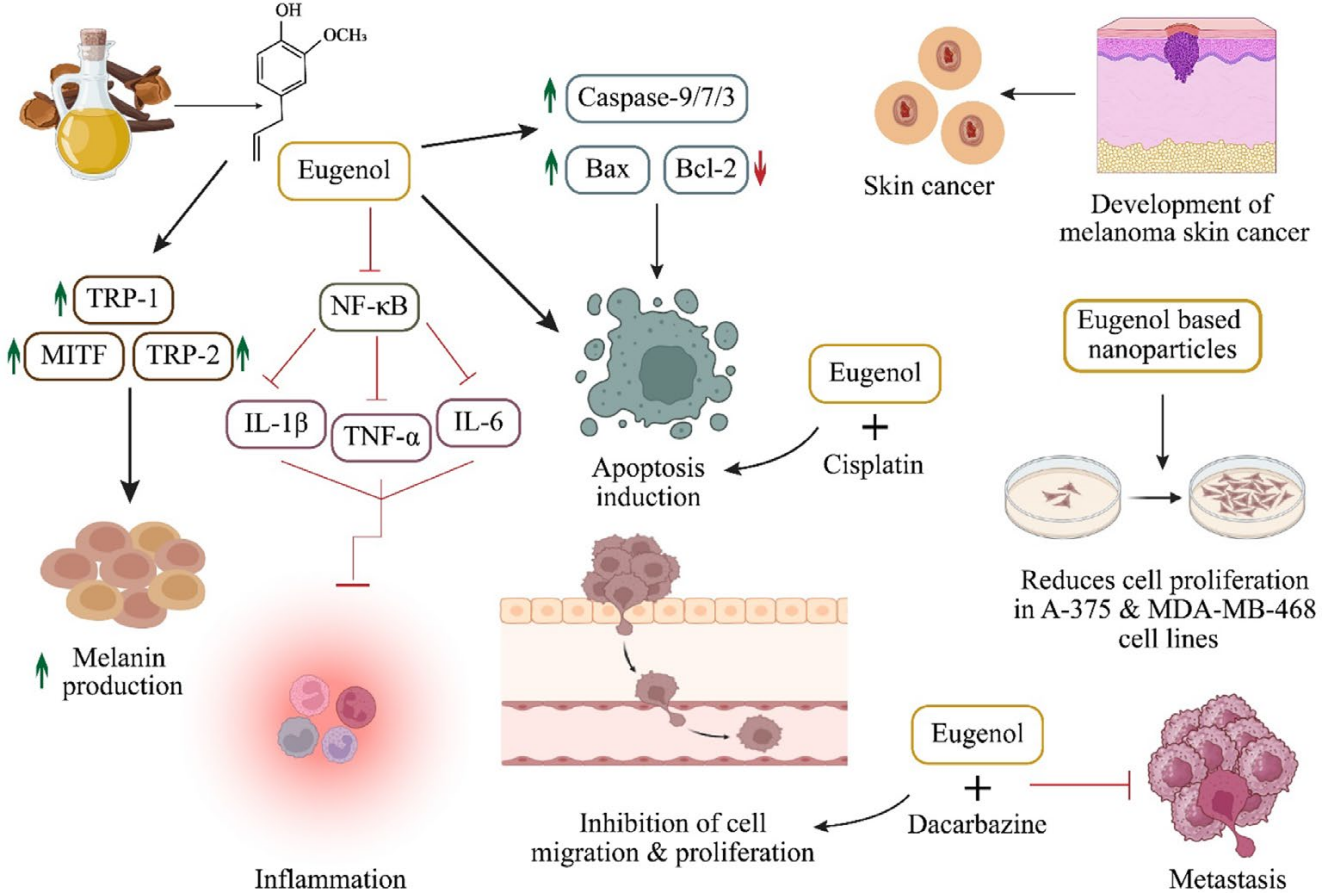
Anticancer potential of eugenol against skin cancer through upregulation of caspase‐9/7/3, Bax, MITF, TRP‐1, and TRP‐2, downregulation of Bcl‐2, modulation of NF‐κB, IL‐1β, TNF‐α, and IL‐6, and inhibition of cell proliferation and migration.

The previous eugenol studies proved its efficiency in protecting melanocytes and effectiveness against melanoma and other skin cancers. Uto et al. (2022) reported eugenol's protective effect in melanocytes through improved melanin production via enhanced TRP‐1, TRP‐2, and MITF expression. Valizadeh et al. (2021) developed eugenol‐based chitosan NPs to mitigate cancer in A‐375 and MDA‐MB‐468 cell lines. They concluded eugenol (73 and 79 μg mL^−1^) significantly reduced cell proliferation. The combined therapy has always proved effective compared with single therapy. Mishra et al. (2019) reported that combined delivery of eugenol and dacarbazine by hyaluronic acid‐coated liposomes inhibited cell migration, proliferation, and metastasis in cancer cells. In a combined therapy, eugenol and chios gum mastic showed apoptotic effects in G361 human melanoma cells. The co‐treatment substantially reduced MMP, Bcl‐2, activated caspase‐9/3/7, and enhanced Bax expression (Jo et al. 2013). In another study, the co‐treatment of eugenol and cisplatin reduced cell proliferation and induced apoptosis in G361 melanoma cells via inhibited MMP and proteasome activity, increased Bax and caspase‐9/7/3 expression, and declined Bcl‐2 expression (Park et al. 2011). Pisano et al. (2007) reported the antiproliferative activity of eugenol‐related biphenyls in melanoma cells via apoptosis induction. Anticancer potential of eugenol against SC via metastasis inhibition, reduction of IL‐6, IL‐1β, TNF‐α, and Bcl‐2 expression, and Bax, caspase‐3/7/9 upregulation is displayed in Figure 4.

### 6.8 Kidney Cancer

Kidneys are involved in metabolism, filtration, absorption, and excretion, and the production of hormones and enzymes. Despite its prime significance, the peril of renal cancer has been alarming the global population. Kidney cancer is the 10th most common cancer in both genders and accounts for ~4%–5% of cases. Renal cell carcinoma (RCC) is the most common type of renal cancer, and nine of 10 renal cancers are RCC. Smoking, obesity/overweight, hypertension (HTN), congenital abnormalities, family history, chronic renal ailments, and exposure to certain chemicals are responsible for cancer development (Scelo and Larose 2018). The RCC pathogenesis starts from the proximal renal tubular epithelium, and structural amendments occur on the short arm of the 3p chromosome. The genes linked with genetic mutations are VHL, BAP‐1, PBRM‐1, SETD2, MTOR, and KDM5C (Hsieh et al. 2017). The PBRM‐1 plays a role in cell cycle regulation and replicative senescence and encodes BAF180 protein, a major contributor to RCC occurrence. Therefore, transformed PBRM‐1 results in an abnormal BAF180 and uncontrolled cell growth and tumorigenesis (Liu et al. 2020).

Nuclear factor erythroid‐2‐related factor 2 (Nrf2) activation is an important factor that regulates the expression of genes vital for redox homeostasis and antioxidant defense. Kuang et al. (2023) studied methyl eugenol's effect on oxidative damage in the kidneys of mice and reported that methyl eugenol (20 mg·kg^−1^·d^−1^, i.p.) significantly improved Nrf2 expression and modulated the AMPK/GSK3β axis. Gamma‐ray exposure can effectively mutate genes and can cause renal damage and carcinoma. Elkady and Ebrahim (2023) reported that eugenol (20 mg/kg, i.p.) enhanced SOD, GSH, and CAT and reduced MDA, IL‐6, IL‐1β, TNF‐α, and serum creatinine in renal‐injured rats. Renal injury can cause oxidative damage and lead to renal cancer. Fathy et al. (2022) stated the nephroprotective potential of eugenol (100 mg/kg) against CCl4‐induced liver damage and OS. Eugenol reduces NOX2, NOX4, IL‐6, TNF‐α, and cyt c and modulates TGF‐β and Akt signaling pathways. Aboelwafa et al. (2022) reported eugenol's nephroprotective effect against silver nanoparticles‐induced renal damage. They concluded that eugenol improved CAT, GSH, SOD, P53, and Caspase‐3 while inhibiting MDA, TNF‐α, IL‐6, and Bcl‐2 expression. Diabetic nephropathy could be one reason to induce oxidative damage and lead to cancer. Garud and Kulkarni (2017) researched that eugenol (5 and 10 mg kg^−1^/day) ameliorated diabetic nephropathy and oxidative damage by reducing TGF‐β1 expression in Sprague–Dawley rats.

### 6.9 Other Cancers

Eugenol has proved beneficial against liver carcinoma through cell cycle arrest, reduced pro‐inflammatory markers, and apoptosis. In a study, eugenol (6.25 and 25 μg/mL) reduced TNF‐α and ROS and enhanced gene expression of GPx and CYP2E1 in HepG2 cell lines. Majeed et al. (2014) reported that eugenol‐loaded nanoemulsions induced apoptosis in HB8065 liver cancer cells. Khaliq et al. (2024) investigated eugenol's anticancer potential in HL‐60 leukemia cell lines and concluded that eugenol (14.1uM) enhanced apoptosis and improved Caspase‐3/9 expression. Arsenic trioxide (As2O3) is a chemotherapeutic drug for leukemia; however, its adverse impact on cardiomyocytes limited its therapeutic effect. Binu et al. (2021) reported that eugenol established protective action against lethal outcomes of As2O3 without affecting its anticancer potential in the HL‐60 cell line. Eugenol proved effective in HL‐60 cell lines by inducing ROS‐mediated apoptosis with a 23.7 IC50 value (Yoo et al. 2005).

Glioma is a fatal disease affecting the central nervous system, causing recurrent primary tumors of glial cells. Li et al. (2020) investigated eugenol‐loaded chitosan polymer against C6 glioma cells and showed that eugenol‐chitosan polymer inhibited NF‐κβ expression and induced apoptosis. Liang et al. (2015) reported eugenol‐induced apoptosis in DBTRG‐05MG human glioma cells. They resulted from eugenol‐induced apoptosis via ROS production and caspase‐9/3 activation. The conjugated molecule of D‐glucose and eugenol (96.2 μg/mL) reduced cell proliferation in K7M2 cells of osteosarcoma (Razak et al. 2021). Shin et al. (2007) stated that eugenol induced cell apoptosis in osteosarcoma cells by activating caspase‐3, DFF‐45 PARP, and lamin A.

The anti‐inflammatory properties of eugenol have boosted its applications in oral health and cancers. Duicu et al. (2018) reported eugenol reduced mitochondrial respiration in SCC‐4 squamous cell carcinoma cell lines and thus proved effective in inhibiting tumor progression. Kim and Park (2015) studied the eugenol effect in HSC‐2 oral squamous carcinoma cells and resulted in apoptosis induction via augmented caspase‐3 and Bak expression. Gynecological malignancies are threatening women's lives and critically affecting life quality due to malignancy adverse consequences. Islam and Aboussekhra (2019) studied the conjugated impact of cisplatin and eugenol against SKOV3 and OV2774 ovarian cancer cell lines. They concluded that combined therapy subdued the Notch‐Hes1 pathway and downregulated drug resistance ABC transporter genes. Table 2 represents in vivo studies on the anticancer potential of eugenol.

**TABLE 2:** In vivo studies on anticancer potential of eugenol.

	Cancer type	Animals	Dose/route/duration	Mechanism	References
Eugenol	Adenocarcinoma	BALB/c mice	100 mg/kg IP	**↓**Tumor growth, **↑**Apoptosis	(Jaganathan et al. 2010)
	Adenocarcinoma	Xenografted nude mice	100 mg/kg 4 weeks	**↓**Tumor growth, NF‐κB, cyclin D1	(Al‐Sharif et al. 2013)
	Lung cancer	strain A mice	100 mL/mouse/day	**↑**caspase 3, Bcl‐2/Bax, **↓**COX‐2, cMyc, Hras	(Banerjee et al. 2006)
	Gastric cancer	Wistar rat	100 mg/kg/3 times/week	**↓**Cell proliferatio, NF‐κB, **↑**cyclin B, cyclin D1, and PCNA	(Manikandan et al. 2010)
	Melanoma	B6D2F1 Mice	125 mg/kg/2/week IP	**↓**Tumor growth, Metastasis	(Ghosh et al. 2005)
	Skin cancer	Swiss mice	30 mL/28 weeks	**↑**Apoptosis, p53, p21WAF1, **↓**iNOS, COX‐2, TNF‐*α*, IL‐6, PGE‐2	(Kaur et al. 2010)
	Skin cancer	Swiss mice	1.25 mg/kg orally	**↓**H‐ras, c‐Myc, Bcl‐2, **↑**Bax, P53 Caspase‐3	(Pal et al. 2010)

## 7 Hypoglycemic Potential

Diabetes mellitus (DM) is a metabolic, endocrine disease affecting 25% of the world population, characterized by elevated blood glucose levels either due to limited insulin synthesis or impaired body response to insulin. Several major risk factors, including obesity, smoking, lifestyle and dietary patterns, inherited aberrations, environmental toxins, and chemicals, are responsible for diabetes (Noman et al. 2025). Diabetes can lead to multiple disorders, including hypertension, stroke, inflammatory bowel disorders (IBD), and cancers. Diabetic neuropathy, nephropathy, retinopathy, and diabetic foot are diabetes‐induced microvascular complications that damage other organs of the body, like the heart, kidney, brain, and legs (Zakir et al. 2023). Diabetes can be categorized into type 1 diabetes mellitus (T1DM), type 2 diabetes mellitus (T2DM), and gestational diabetes mellitus (GDM). The types vary according to their risk factors and pathophysiology, as T1DM is linked with immune‐mediated β‐cell dysfunction and can be diagnosed by GAD65 autoantibody (Kahaly and Hansen 2016). T2DM involves insulin resistance and reduced insulin sensitivity in muscle, liver, and adipose tissue, leading to hyperinsulinemia, overactivity of pancreatic β‐cells, and eventually β‐cell destruction (Wondmkun 2020). GDM is associated with pregnancy and develops during the 3rd trimester due to hormonal dysregulation, nutrient accumulation, and obesity (Banday et al. 2020).

The studies on the antidiabetic potential of eugenol have proved its hypoglycemic and hypolipidemic activity. Chilukoti et al. (2024) verified the antidiabetic activity of eugenol in rats. They concluded that eugenol (400 mg/kg) significantly lowered glucose levels, reduced OS and inflammation, inhibited MDA levels, and improved GSH. DM is often accompanied by oxidative damage and apoptosis. In this context, Jiang et al. (2024) studied eugenol's protective role against T1DM‐induced OS and showed that eugenol supplementation activated Nrf2, upregulated NQO‐1 and HO‐1, mitigated β cell damage, and reduced OS‐linked cell apoptosis. High‐fat diet consumption is one major cause of DM and abnormal lipid levels. Jiang et al. (2022) studied the antidiabetic effect of eugenol in high‐fat diet/streptomycin‐induced diabetic mice. The study showed that eugenol increased GLUT4 translocation and AMPK phosphorylation in skeletal muscles, increased intracellular Ca2+ levels via TRPV1, and activated CaMKK2. Overall, eugenol has been found efficient against high‐fat‐induced diabetes. The complications of DM include reproductive system dysfunction and infertility in both males and females. Kokabiyan et al. (2023) illuminated eugenol's role in lipid profile reduction, OS alleviation, and protection against liver and ovary injury. They found that eugenol (12 and 24 mg/kg) diminished high levels of total cholesterol, triglycerides, LDL, and liver enzymes (ALT, AST, and ALP). Furthermore, eugenol reduced PPAR‐α and COX‐2 expression and attenuated diabetes.

## 8 Cardioprotective Activity

The heart is one of the vital organs involved in the blood supply to all organs. Therefore, the disturbance in cardiac function leads to reduced blood supply to other body parts, and even blockage in cardiac vessels results in poor blood supply to the heart and eventually causes cardiac dysfunction or cardiac failure. Studies have reported that eugenol is used as an anesthetic due to its suppressive effects on voltage‐gated Na + channels (Nav), which are expressed in nociceptive neurons. Teixeira‐Fonseca et al. (2021) investigated eugenol's impact on arrhythmias via interacting cardiac sodium channels. They found that eugenol caused negative inotropic and chronotropic effects in atria, decreased Na concentration, and blocked channels in the inactivated state. Chemical toxins are linked with disrupting biochemical reactions and induce oxidative damage to vital organs. Chlorpyrifos, an organophosphate toxin used in agriculture, can cause OS, apoptosis in several body tissues, including the heart, and genetic mutation. Eugenol (250 mg/kg BW) was tested against chlorpyrifos‐induced genotoxicity and cardiac damage in rats. The findings showed that eugenol substantially diminished the expression of SERCA2a and NKX2‐5 genes in cardiac tissue, thus regulating heart function and growth (Ranjbar et al. 2021). A study on animal eugenol inhalation effectively reduced psychological stress and cortisol levels. Considering this, Yamanashi et al. (2020) studied the eugenol inhalation effect in humans and reported that eugenol reduced salivary cortisol levels and brain stress in healthy adult males. Fen et al. (2018) reported that eugenol (20 mg/kg/day) prevents the heart against ischemia/reperfusion injury in rats via reduced myocardial MDA, serum cardiac troponin I, creatine kinase‐MB, TNF‐α, IL‐6 levels, and attenuated myocardial injury. Furthermore, eugenol reduced BAX and caspase‐3 expression while improving B‐cell lymphoma 2 expression. Mnafgui et al. (2016) investigated the antithrombotic and preventive effects of eugenol against isoproterenol‐induced myocardial infarction in rats. They reported that eugenol (50 mg/kg) reduced troponin‐T, CK‐MB, LDH, and ALT levels, mitigated myocardium necrosis, and inhibited plasma inflammatory biomarkers (γ globulins, α1, α2, β1, β2, and fibrinogen).

## 9 Eugenol and Pulmonary Disorders

The pulmonary system, called the respiratory system, mainly consists of the nose, trachea, and lungs working together to bring oxygen into the body and remove carbon dioxide. Lungs are prime vulnerable organs in the pulmonary system protected by the rib cage. Cigarette smoking, environmental toxins, and pathogenic infections are major causes of lung injury. Moreover, pulmonary thromboembolism could be a potent risk factor for lung damage. Huang et al. (2024) studied the antiplatelet activity of eugenol in humans and rats and reported that eugenol (2 μM) concentration inhibited collagen and arachidonic acid‐induced platelet aggregation. They concluded that eugenol inhibited platelet activation by suppressing PLCγ2–PKC and cPLA2–TxA2 cascade. Chronic obstructive pulmonary disease is characterized by diffuse chronic lung inflammation and alveolar destruction. Eugenol and dimmer biseugenol (20 mg/kg) alleviated elastase‐induced emphysema, alveolar destruction, and inflammation in C57BL/6 mice via modulating MMP‐9, NF‐kB, and TIMP‐1 and reducing iNOS (Taguchi et al. 2023). Barbosa‐de‐Oliveira et al. (2023) studied eugenol's protective effect against cigarette smoke‐induced acute lung injury in C57BL/6 mice through reduced pro‐inflammatory cytokines while increasing antioxidant enzymes and anti‐inflammatory markers. Tao et al. (2022) studied aspirin eugenol ester (AEE) protective effect in rats against LPS‐induced lung injury. They concluded that AEE (54, 108, and 216 mg kg^−1^) inhibited MDA, MPO, CRP, LDH, TNF‐α, IL‐6, and IL‐1β levels, whereas it improved GSH, SOD, CAT, and GPx activity. In another study, aspirin (150 mg/kg) significantly reduced TNF‐α, IL‐1β, IL‐6, and protein oxidation while improving SOD, GSH, and CAT activity in LPS‐induced lung injury in BALB/c mice (Magalhães et al. 2019).

## 10 Antimicrobial Properties

The antimicrobial activity of eugenol has been widely reported. Di Consiglio et al. (2023) formulated eugenol and hydroxyethyl methacrylate‐based polymers, investigated them against *S. epidermidis*, and reported the significant antibacterial potential of the eugenol‐based polymer. Similarly, Qian et al. (2020) investigated the antimicrobial activity of eugenol against *K. pneumoniae* and reported that the eugenol minimum inhibitory concentration (MIC) was 0.2 mg/mL. In addition, eugenol damaged the cell membrane of *K. pneumoniae*, reduced cell membrane hyperpolarization, and increased membrane permeability. The antimicrobial activity of eugenol against various bacterial and fungal strains is highlighted in Table 3.

**TABLE 3:** Antimicrobial of eugenol against various bacterial and fungal strains.

	Microorganism	Strains	MIC range	References
Eugenol	Bacteria	*S. flexneri*	0.5, 0.8 mg/mL	(Bai et al. 2022)
		*S. aureus* , *E. coli* , *K. pneumonia* , *A. baumannii*	8.5, 12.75, 17 mg/mL	(Shahabadi et al. 2019)
		*S. sonnei*	0.5 mg/mL	(Su et al. 2022)
		*V. parahaemolyticus*	0.1%–0.6%	(Ashrafudoulla et al. 2020)
	Fungi	*F. solani*	0.5 and 9.04 μg mL^−1^	(Maximino et al. 2020)
		*R. solani*	200 μg ml^−1^	(Zhao et al. 2021)
		*Z. rouxii*	0.4 μL/mL	(Cai et al. 2019)
		*C. neoformans*	125, 500 μg/mL	(Hassanpour et al. 2020)

## 11 Neuroprotective Effect

The nervous system (NS) comprises the central nervous system (CNS), and the peripheral nervous system (PNS) is a complex system that receives, processes, and responds to sensory information. The CNS covers the brain and spinal cord and works along with the PNS nerve network. Several neurodegenerative disorders, including Alzheimer's disease, dementia, epilepsy, depression, and anxiety, affect people of all ages (Xu et al. 2022). The neuroprotective potential of eugenol and its efficacy against brain disorders have been reported in previous studies. Zanikov et al. (2023) studied the combined neuroprotective effect of psilocybin and eugenol (1:10, 1:20, or 1:50) against LPS‐induced brain inflammation in mice by reducing COX‐2, TNF‐α, IL‐1β, and IL‐6 expression. Traumatic brain injury (TBI) results in demise or long‐term functional incapacities. Barot and Saxena (2021) reported that eugenol (25, 50, and 100 mg/kg/day) for 7 days reduced TBI, lipid peroxidation of brain tissue, and edema. Overall, eugenol ameliorated the neurochemical and behavioral outcomes of trauma. Eugenol ameliorated aluminum‐induced neurotoxicity in rats and reduced oxidative damage and inflammation in Wistar (Okpanachi 2023). El‐Far et al. (2022) stated that eugenol, combined with other bioactive compounds, proved more effective in mitigating d‐galactose‐induced aging‐related oxidative alterations in the brains of rats. They showed that eugenol (10, 20 mg/kg/day) and carvacrol (40, 80 mg/kg/day) upregulated p53 and p21 expression, modulated CPK and TAG levels, and enhanced brain antioxidant capacity. In another study, eugenol improved dopamine activation in PC12 cells, thus proving beneficial against Parkinson's disease (Pavan et al. 2023).

## 12 Eugenol and Gut Health

Gut health is one of the major challenges because numerous factors, including pathogenic infection, asymmetrical eating patterns, pesticide residues, toxins, and chemicals, are contributing to gut disorders. Furthermore, disturbed gut results in dysbiosis, an imbalance between probiotics in the colon, consequently leading to inflammation and other chronic problems (Parkin et al. 2021). Eugenol has been proven effective in modulating gut microbiota and attenuating adiposity in high‐fat diet‐fed C57BL/6J mice. Additionally, eugenol reduced gut dysbiosis and improved *Firmicutes*, *Dubosiella*, and *Blautia* spp., while reducing *Desulfobacterota, Alistipes*, and *Bilophila* spp. (Li, Yuan, et al. 2022). Rodrigues et al. (2022) studied the beneficial impact of eugenol supplementation on gut health in high‐fat‐fed C57BL/6 mice. The study's findings proved that Eug (500 mg kg^−1^) for 8 weeks enhanced *Actinobacteria*, reduced *Proteobacteria*, and decreased hepatic lipid accumulation in mice. The gut‐brain axis (GBA) is the two‐way communication system that links brain cognitive centers with intestinal functions, and gut microbiota plays a significant role in GBA. Li, Zhao, et al. (2022) reported that eugenol ameliorated FLD in rats through GBA involving glucagon‐like Peptide‐1. They found that 8 weeks of eugenol administration in rats reduced serum, hepatic triglycerides, and total cholesterol. In addition, eugenol promoted GLP‐1 secretion and augmented c‐fos expression. Silva Júnior et al. (2020) reported that eugenol, thymol, and piperine improved gut health and nutrient digestibility in weaned piglets. Hobani et al. (2022) demonstrated the gastroprotective and anti‐inflammatory effects of eugenol against ethanol‐induced toxicity in rats. Eugenol decreased ulcer index via declined NO, TNF‐α, and IL‐6 concentration and enhanced GSH and PGE2 levels. Moreover, the eugenol administration upregulated HSP70 and downregulated iNOS expression.

## 13 Eugenol and Delivery Systems

Eugenol exhibits various therapeutic properties such as antioxidant, anti‐inflammatory, and anticancer properties. However, its therapeutic potential is limited by poor solubility and stability. Multiple delivery systems, such as liposomes, nanoparticles, nanoemulsions, and hydrogels, enhance its bioavailability, controlled release, and targeted delivery, making eugenol more effective for pharmaceutical and biomedical applications. Considering this, nanoparticles are a suitable and promising approach for drug delivery. In a recent study, Q0‐eugenol nanoemulsion was developed to evaluate its anticancer potential against BC and HCC cell lines. The findings showed that Q0‐eugenol nanoemulsion exhibited an antiproliferative effect and induced apoptosis (16.8%) in PLC/PRF/5 cell lines, whereas the apoptosis rate was 19% in KPL1 cell lines. Overall, the Q0‐eugenol nanoemulsion proved potent against cancer cell lines and reduced proliferation via ROS‐mediated apoptosis (Abbasi et al. 2025). In another study, eugenol‐loaded nano delivery system (EUG@CMC‐PGMA‐CS) was investigated for antibacterial and insect repellent activity. It was observed that this nano‐delivery system exhibited strong insect repellent and antibacterial activity against *Spodoptera litura* and *S. aureus* , respectively (Zuo et al. 2024). Moreover, eugenol extracted from *Syzygium aromaticum* was used to prepare a gel for the treatment of atopic dermatitis (AD), and eugenol‐loaded transethosomes gel was synthesized by using Carbopol 940. The results exhibited that eugenol‐loaded gel significantly enhanced the retention of the drug in the skin. Transethosomal gel was able to reduce IL‐6 levels, ear thickness, differential leukocyte count (DLC), and TLC in the Swiss albino mice model of AD (Kashyap et al. 2024). A ketoconazole (KTZ) and eugenol‐based nanoemulsion was developed for topical delivery against *Candida albicans* . The ex vivo retention studies showed the accumulation of nanoemulsion at different layers of the skin when applied topically. In addition, the cytotoxicity of the developed nanoemulsion in human HaCaT cells revealed the utility of this nanocarrier in reducing the cell toxicity of KTZ. The higher antifungal activities of nanoemulsion at 19.23‐fold lower concentrations for planktonic growth (Dubey et al. 2024).

## 14 Safety and Food Applications

Eugenol has been safe for consumption within the recommended dose; however, toxicity reports have been documented. The World Health Organization (WHO) specified the acceptable daily intake (ADI) of eugenol, which is 2.5 mg/kg body weight. Eugenol by‐products and end metabolites have been associated with toxicity, as quinone methide produced by oxidation of eugenol is toxic to hepatocytes. The lipophilic nature of eugenol results in cell damage, and the pro‐oxidant activity of eugenol triggers the production of ROS, which contributes to tissue damage. Moreover, eugenol binds to lysine, leading to protein deactivation and toxicity (Özbek and Ergönül 2022). The antimicrobial potential of eugenol makes it a suitable candidate for food applications. Orlo et al. (2023) developed eugenol‐based antimicrobial coatings and tested them against foodborne pathogens and spoilage microbes. They found the highest antimicrobial activity of these coatings against *S. aureus* . Likewise, Requena et al. (2017) synthesized eugenol carvacrol‐based food packaging films and tested them against foodborne pathogens. Prasetya and Sarjono (2019) examined the antimicrobial potential of polyeugenol (~10 kg mol^−1^) against *E. coli* and *S. aureus* . Polyeugenol (~800–2200 kg mol^−1^) was effective against *E. coli* and *S. aureus* (Rahim et al. 2020).

## 15 Conclusion and Future Perspectives

Eugenol is a key phenylpropene, mainly found in various herbs with a yellowish appearance and lipophilic nature. The hydrophobic and volatile nature of eugenol makes it less bioavailable and makes its absorbance difficult however, eugenol derivatives have more bioavailability due to stability. The strong antioxidant potential of eugenol provides it with anti‐inflammatory effect, which proves effective in the reduction of ROS, RNS, and cancer prevention. Anticancer‐based studies proved its effectiveness against lung, breast, colorectal, cervical, leukemias, gliomas, and melanoma. Studies have proved that it can inhibit cell proliferation, induce apoptosis, reduce pro‐inflammatory markers, improve antioxidant enzymes (GSH, GPx, SOD, CAT) and modulate oncogenes and TSGs. Furthermore, the in vitro studies have demonstrated that it has the potential to induce cell cycle arrest. Besides its anticancer potential, it contains hypoglycemic and antimicrobial activity, protects the heart, lungs, and brain against various ailments, and also improves gut health. Above all, eugenol is a remarkable bioactive component with antioxidant, anticancer potential, and other bioactivities. Moreover, advances in nanoformulations and targeted delivery systems may significantly improve its bioavailability and specificity. Additionally, eugenol's synergistic effects with other phytochemicals and its potential to reduce multidrug resistance open new avenues for combination therapies. Future research should focus on clinical trials regarding cancer and other health anomalies management; detailed pharmacokinetics and interaction with molecular targets at a genomic and proteomic level will help develop more precise cancer treatment strategies.

## Author Contributions

**Ahmad Mujtaba Noman:** conceptualization (equal), writing – original draft (equal). **Muhammad Tauseef Sultan:** conceptualization (equal), writing – original draft (equal). **Aimen Mazhar:** investigation (equal), writing – original draft (equal). **Waqas Ahmad Khan:** data curation (equal), writing – original draft (equal). **Muhammad Imran:** data curation (equal), resources (equal). **Muzzamal Hussain:** software (equal), supervision (equal), writing – review and editing (equal). **Ehab M. Mostafa:** data curation (equal), visualization (equal). **Ahmed H. El‐Ghorab:** investigation (equal), validation (equal). **Mohammed M. Ghoneim:** supervision (equal), validation (equal). **Samy Selim:** project administration (equal), writing – review and editing (equal). **Mohamed A. Abdelgawad:** project administration (equal), validation (equal), visualization (equal). **Entessar Al Jbawi:** data curation (equal), supervision (equal). **Suliman A. Alsagaby:** writing – review and editing (equal). **Waleed Al Abdulmonem:** data curation (equal), investigation (equal).

## Conflicts of Interest

The authors declare no conflicts of interest.

## Data Availability

The data that support the findings of this study are available from the corresponding author upon reasonable request.
